# Measuring Rubisco activity: challenges and opportunities of NADH-linked microtiter plate-based and ^14^C-based assays

**DOI:** 10.1093/jxb/eraa289

**Published:** 2020-06-30

**Authors:** Cristina R G Sales, Anabela Bernardes da Silva, Elizabete Carmo-Silva

**Affiliations:** 1 Lancaster Environment Centre, Lancaster University, Library Avenue, Lancaster, UK; 2 BioISI - Biosystems & Integrative Sciences Institute, Faculty of Sciences, University of Lisbon, Lisbon, Portugal; 3 Australian National University, Australia

**Keywords:** Crop improvement, dPGM, enzyme activity assay, GAPDH–GlyPDH, NADH, PEPC–MDH, plant phenotyping, PK–LDH, Rubisco, wheat

## Abstract

Rubisco is central to carbon assimilation, and efforts to improve the efficiency and sustainability of crop production have spurred interest in phenotyping Rubisco activity. We tested the hypothesis that microtiter plate-based methods provide comparable results to those obtained with the radiometric assay that measures the incorporation of ^14^CO_2_ into 3-phosphoglycerate (3-PGA). Three NADH-linked assays were tested that use alternative coupling enzymes: glyceraldehyde-3-phosphate dehydrogenase (GAPDH) and glycerolphosphate dehydrogenase (GlyPDH); phosphoenolpyruvate carboxylase (PEPC) and malate dehydrogenase (MDH); and pyruvate kinase (PK) and lactate dehydrogenase (LDH). To date there has been no thorough evaluation of their reliability by comparison with the ^14^C-based method. The three NADH-linked assays were used in parallel to estimate (i) the 3-PGA concentration–response curve of NADH oxidation, (ii) the Michaelis–Menten constant for ribulose-1,5-bisphosphate, (iii) fully active and inhibited Rubisco activities, and (iv) Rubisco initial and total activities in fully illuminated and shaded leaves. All three methods correlated strongly with the ^14^C-based method, and the PK–LDH method showed a strong correlation and was the cheapest method. PEPC–MDH would be a suitable option for situations in which ADP/ATP might interfere with the assay. GAPDH–GlyPDH proved more laborious than the other methods. Thus, we recommend the PK–LDH method as a reliable, cheaper, and higher throughput method to phenotype Rubisco activity for crop improvement efforts.

## Teaser

Measurement of Rubisco activity by NADH-linked microtiter plate-based assays produces reliable results for economical and reasonably high throughput plant phenotyping provided that a few critical steps are taken into consideration.

## Introduction

Rubisco is the most abundant protein in nature and catalyses CO_2_ fixation via the carboxylation of ribulose-1,5-bisphosphate (RuBP) (Ellis, 1979). However, Rubisco is catalytically inefficient and is commonly the limiting step of CO_2_ assimilation (Parry *et al.*, 2013). Its inefficiencies include a slow turnover rate and the ability to fix O_2_ instead of CO_2_, which leads to the consumption of energy and release of fixed CO_2_ (Tcherkez, 2016). Improving Rubisco’s properties and regulation would lead to increased photosynthetic efficiency in crop plants (Carmo-Silva *et al.*, 2015; Long *et al.*, 2015; Betti *et al.*, 2016), and a number of efforts have been made to engineer Rubisco (reviewed by Sharwood, 2017). Catalytic diversity in Rubisco’s properties has been established in a range of crop and wild species (Hermida-Carrera *et al.*, 2016; Orr *et al.*, 2016; Prins *et al.*, 2016; Sharwood *et al.*, 2016*a*). However, phenotyping of diversity in Rubisco activity to inform breeding of better crops requires reliable, economical and reasonably high throughput methods.

Prior to catalysing the carboxylation/oxygenation of RuBP, Rubisco must be carbamylated. The catalytic sites of the enzyme (E) are activated by carbamylation via the reversible binding of non-substrate CO_2_ (C) to the amino group of a conserved lysine residue, and the carbamate is subsequently stabilized by the rapid binding of Mg^2+^ (ECM). Binding and enolization of the substrate RuBP to ECM precedes the fixation of CO_2_ or O_2_. A number of sugar phosphate derivatives bind tightly to the catalytic sites of Rubisco before (EI) or after (ECMI) carbamylation, preventing carbamylation and/or substrate binding, respectively (Cleland *et al.*, 1998). Binding of the substrate RuBP to the uncarbamylated enzyme (ER) also prevents carbamylation and causes inhibition of Rubisco activity. The removal of tightly bound inhibitors from carbamylated and decarbamylated Rubisco catalytic sites is dependent on interaction with Rubisco activase. Rubisco catalytic sites free from inhibitory compounds become available for carbamylation and/or to participate in catalysis (for a review on Rubisco inhibitors see Parry *et al.*, 2008). The extent of carbamylation of Rubisco *in vivo* can be assessed by comparing the initial activity, determined immediately upon protein extraction, with the total activity, determined after incubation of the leaf extract with CO_2_ and Mg^2+^. The last step allows the carbamylation of available catalytic sites (Parry *et al.*, 1997, 2008).

Rubisco activities and activation state can be reliably and precisely determined by a radiometric assay that measures the incorporation of ^14^CO_2_ to monitor the production of the acid-stable product, 3-phosphoglycerate (3-PGA) (Lorimer *et al.*, 1977; Parry *et al.*, 1997). Routinely used since the 1970s, the radiometric assay is highly accurate and specific, but depends on hazardous substances and rigorous safety measures for using radiochemicals, limiting its application. Spectrophotometric assays use a number of additional enzymes to couple the production of 3-PGA to a change in absorbance, typically associated with oxidation of NADH. These assays provide a convenient alternative for measuring Rubisco activity since there is no requirement for specialized facilities associated with using radioactive substances, the reaction rate is immediately observed enabling adjustment of the assay conditions, and the use of consumables can be scaled down to provide a cheaper and environmentally friendly option compared with radiometric assays (Ward and Keys, 1989; Scales *et al.*, 2014).

Whether the spectrophotometric and the radiometric assays give equivalent results for Rubisco activity and the catalytic properties remains unclear. A number of studies have compared the different methods (Anderson and Fuller, 1969; Ward and Keys, 1989; Reid *et al.*, 1997; Sulpice *et al.*, 2007; Sharwood *et al.*, 2016*b*), and most of these used the first spectrophotometric assay reported for Rubisco activity, which uses glyceraldehyde-3-phosphate dehydrogenase (GAPDH) and glycerolphosphate dehydrogenase (GlyPDH) as a coupling enzyme (Racker, 1957, 1962). The outcomes from the different comparisons have been inconsistent, with some studies finding the same results for Rubisco activity measured by spectrophotometric and radiometric assays (Ward and Keys, 1989; Sulpice *et al.*, 2007), and others showing lower values when measured by NADH-linked assays compared with the ^14^CO_2_ fixation assay (Reid *et al.*, 1997; Sharwood *et al.*, 2016*b*).

Most studies employing the NADH-linked assay for measuring Rubisco activity utilize cuvettes to measure the change in absorbance in a spectrophotometer (Racker, 1962; Anderson and Fuller, 1969; Lilley and Walker, 1974; Ward and Keys, 1989; Lan and Mott, 1991; Du *et al.*, 1996; Reid *et al.*, 1997; Kubien *et al.*, 2011; Sharwood *et al.*, 2016*b*). Adaptation of the method to a microtiter plate format considerably reduces the assay volume and enables higher throughput (Sulpice *et al.*, 2007; Scales *et al.*, 2014). However, microtiter plate-based assays may decrease the accuracy of the results, for example due to inaccuracies associated with pathlength correction of the measured absorbance, as this depends on the volume and solution used in the assay (Lampinen *et al.*, 2012).

**Fig. 1.**
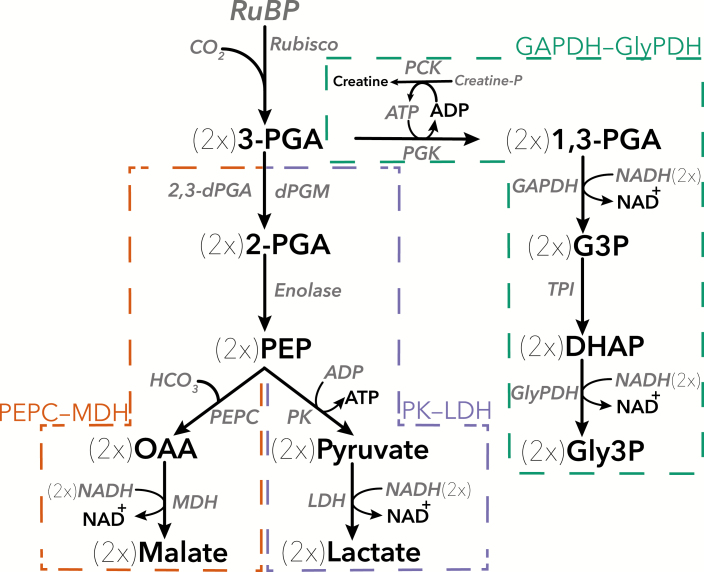
Three alternative NADH-linked spectrophotometric assays using different coupling enzymes for measuring Rubisco activity: PEPC–MDH (left), PK–LDH (center) and GAPDH–GlyPDH (right). See text for detailed description of each protocol. 1,3-PGA, 1,3-diphosphoglycerate; 2-PGA, 2-phosphoglycerate; 2,3-dPGA, 2,3-diphospho-D-glyceric acid; 3-PGA, 3-phosphoglycerate; DHAP, dihydroxyacetone phosphate; dPGM, 2,3-dPGA-dependent phosphoglycerate mutase; GAPDH, glyceraldehyde-3-phosphate dehydrogenase; G3P, glyceraldehyde-3-phosphate; Gly3P, glycerol 3-phosphate; GlyPDH, glycerolphosphate dehydrogenase; LDH, lactate dehydrogenase; MDH, malate dehydrogenase; OAA, oxaloacetate; PCK, creatine phosphokinase; PEP, phosphoenolpyruvate; PEPC, phosphoenolpyruvate carboxylase; PGK, 3-phosphoglycerate kinase; PK, pyruvate kinase; RuBP, ribulose-1,5-bisphosphate; TPI, triosephosphate isomerase.

Three NADH-linked assays have been described that use a different set of coupling enzymes to measure Rubisco initial and total activity and activation state in a spectrophotometer (Fig. 1). These assays use five reactions to couple RuBP carboxylation and 3-PGA formation to NADH oxidation. Here, we refer to these assays as GAPDH–GlyPDH (based on Kubien *et al.*, 2011), phosphoenolpyruvate carboxylase (PEPC)–malate dehydrogenase (MDH), and pyruvate kinase (PK)–lactate dehydrogenase (LDH) (both based on Scales *et al.*, 2014). In the GAPDH–GlyPDH assay, 3-PGA is phosphorylated to 1,3-PGA by 3-PGA kinase (PGK) at the expense of ATP; 1,3-PGA is reduced to glyceraldehyde-3-phosphate (G3P) coupled with NADH oxidation by GAPDH; triosephosphate isomerase (TPI) produces dihydroxyacetone phosphate (DHAP) from G3P; and GlyPDH reduces DHAP forming glycerol 3-phosphate (Gly3P) with NADH oxidation. The PEPC–MDH and PK–LDH assays share the first three reactions: RuBP is carboxylated and 3-PGA is formed; 3-PGA is converted to 2-phosphoglycerate (2-PGA) by 2,3-dPGA-dependent phosphoglycerate mutase (dPGM); and enolase converts 2-PGA to phosphoenolpyruvate (PEP). The final two reactions differ between the two assays. In the PEPC–MDH assay, PEPC catalyses the carboxylation of PEP to oxaloacetate (OAA), then OAA is reduced to malate by MDH in a reaction coupled to the oxidation of NADH to NAD^+^. In the PK–LDH assay, PK uses PEP as a substrate, leading to the formation of pyruvate with use of ADP, then pyruvate is reduced to lactate by LDH while NADH is oxidized to NAD^+^. Rubisco produces two molecules of 3-PGA for each RuBP carboxylated. In the PEPC–MDH and PK–LDH assays two NADH are oxidized, while in the GAPDH–GlyPDH assay four NADH are oxidized per RuBP carboxylation. The resulting decrease in NADH concentration can be monitored by measuring the absorbance of the assay solution at 340 nm in a spectrophotometer and adapted to use flat-bottom 96-well microtiter plates to enhance throughput and reduce costs.

We tested the hypothesis that the three NADH-linked microtiter plate-based assays for measuring Rubisco activity and activation state provide comparable results to each other and to the radiometric ^14^C assay. The three photometric assays were used in parallel for a range of applications, providing consistent and reliable results. The challenges, costs, advantages, and disadvantages of using each assay for measuring Rubisco activity are discussed.

## Materials and methods

Detailed protocols for the three different NADH-linked microtiter plate-based assays, the ^14^C-based assay and purification of dPGM are available as a collection in protocols.io dx.doi.org/10.17504/protocols.io.bf8djrs6. The microtiter plates were clear flat-bottom immune non-sterile 96-well-plates (Thermo Fisher Scientific, Waltham, MA, USA), and the microplate reader was a SpectroStarNano (BMG Labtec, Aylesbury, UK).

### Materials

Ultrapure water and high-grade reagents were used. The chemicals were obtained from Sigma-Aldrich (St Louis, MO, USA), except for RuBP (synthesized according to Wong *et al.*, 1980) and dPGM (prepared based on Fraser *et al.*, 1999 and Scales *et al.*, 2014; protocol available at protocols.io dx.doi.org/10.17504/protocols.io.bf8djrs6).

### Rubisco purification

Seeds of *Triticum aestivum* cv. Cadenza (wheat) were sown in trays containing commercial compost mix (Petersfield Growing Medium, Leicester, UK), in semi-controlled conditions at 26/18 °C day/night with a photoperiod of 16 h, and natural light supplemented to maintain a minimum level of 500 μmol photons m^−2^ s^−1^. Leaf material was harvested from ~10 cm high seedlings 2–3 h after the start of the photoperiod, frozen in liquid N_2_ and stored at −80 °C. Rubisco purification from wheat leaves was performed as described by Carmo-Silva *et al.* (2011).

### Fully illuminated and shaded leaf sampling

Seeds of *Triticum aestivum* cv. Cadenza were sown in 2 liter pots containing commercial compost mix (Petersfield Growing Medium). The plants were grown in the same semi-controlled conditions described above. Flag leaves were sampled when plants reached booting (Zadoks stage 4.0–4.5; Zadoks *et al.*, 1974). Samples were collected from fully illuminated leaves exposed to a photosynthetic photon flux density (PPFD) of 625±121 μmol photons m^−2^ s^−1^, and frozen in liquid N_2_ while still illuminated. Samples from shaded leaves were collected after exposure for at least 1 h to a very low PPFD of 5±6 μmol photons m^−2^ s^−1^ and sampled while still in the shade. Measurements of leaf widths were taken to calculate the area of the leaf section (2–5 cm^2^). Leaves were stored at −80 °C until analysis.

### Rubisco activity assays

These protocols are available at protocols.io dx.doi.org/10.17504/protocols.io.bf8djrs6. To test the reliability of each assay in determining differences in Rubisco activity, measurements were performed at 30 °C with fully carbamylated Rubisco (ECM), and with inhibited Rubisco (ER) prepared according to Barta *et al.* (2011). The Rubisco concentration in the assays was 15 μg ml^−1^ for purified enzyme and between 10 and 40 μg ml^−1^ for non-purified enzyme. Rubisco amounts above these values may limit the sensitivity of the NADH-linked assays. Considering that Rubisco can contribute up to 50% of the total soluble protein (TSP) in C_3_ plants and 25% in C_4_ plants (Carmo-Silva *et al.*, 2015), and that 5 μl of extract was added in the reaction, the recommended maximum TSP in leaf samples should be 3.5 mg ml^−1^ for C_3_ plants and double for C_4_ species. By way of example, the measured rate of RuBP consumption reached saturation in wheat leaf extracts containing TSP concentrations above 4 mg ml^−1^ and high Rubisco activity (see Supplementary Fig. S1 at *JXB* online). This high limit of TSP concentration also applies to ^14^C-based assays and tests for each experiment are recommended to ensure that none of the reagents limits the reaction.

Leaf extracts were prepared according to Carmo-Silva *et al.* (2017) with slight modifications. Samples previously stored at −80 °C were ground in an ice-cold mortar and pestle with 1500 μl of extraction buffer containing 50 mM Bicine–NaOH, pH 8.2, 20 mM MgCl_2_, 1 mM EDTA, 2 mM benzamidine, 5 mM ε-aminocaproic acid, 50 mM 2-mercaptoethanol, 10 mM dithiothreitol, 1% (v/v) protease inhibitor cocktail (Sigma-Aldrich) and 1 mM phenylmethylsulfonyl fluoride. The homogenate was clarified by centrifugation at 14 000 *g* and 4 °C for 1 min. The supernatant was immediately used for measuring Rubisco activity at 30 °C. For initial activity, the reaction was started by adding the leaf extract to the complete assay buffer (see below and protocols.io dx.doi.org/10.17504/protocols.io.bf8djrs6 for components of the assay buffer in the different methods). For total activity, the Rubisco in leaf extracts was first activated by incubation in the assay buffer containing CO_2_ and Mg^2+^ in the absence of RuBP for 3 min, and the reaction initiated by adding RuBP.

For the radiometric assay, Rubisco (typically 5–40 μg) was added to the assay buffer (final volume 500 μl) containing 100 mM Bicine–NaOH pH 8.2, 20 mM MgCl_2_, 10 mM NaH^14^CO_3_ (9.25 kBq μmol^−1^), and 0.4 mM RuBP. Reactions were quenched after 30 s by adding 100 μl of 10 M formic acid. Acid-stable ^14^C was determined as in Carmo-Silva *et al.* (2017).

For the GAPDH–GlyPDH NADH-linked assay (Kubien *et al.*, 2011), Rubisco was added to the assay buffer (final volume 200 μl) containing 100 mM Bicine–NaOH pH 8.2, 20 mM MgCl_2_, 10 mM NaHCO_3_, 5 mM DTT, 1 mM ATP, 5 mM phosphocreatine, 0.4 mM NADH, 25 U ml^−1^ creatine phosphokinase, 25 U ml^−1^ GAPDH, 25 U ml^−1^ PGK, 200 U ml^−1^ TPI, 20 U ml^−1^ GlyPDH, and 0.6 mM RuBP.

For the PEPC–MDH NADH-linked assay (Scales *et al.*, 2014), Rubisco was added to the assay buffer (final volume 200 μl) containing 100 mM Bicine–NaOH pH 8.2, 20 mM MgCl_2_, 10 mM NaHCO_3_, 20 mM KCl, 5 mM DTT, 0.2 mM 2,3-diphospho-D-glyceric acid (2,3-dPGA), 0.4 mM NADH, 5 U ml^−1^ enolase, 3.75 U ml^−1^ dPGM, 3.75 U ml^−1^ PEPC, 5 U ml^−1^ MDH, and 0.6 mM RuBP.

The enzymes TPI and PGK, used in the GAPDH–GlyPDH assay, and MDH, used in the PEPC–MDH assay, are provided in ammonium sulfate suspension and it is essential to perform a buffer exchange using a centrifugation filter (Amicon, molecular mass cut-off of 10 kDa, Sigma-Aldrich). The presence of ammonium sulfate in the reactions would otherwise interfere with the assays and decrease the rates estimated via these assays.

For the PK–LDH photometric assay (Scales *et al.*, 2014), Rubisco was added to the assay buffer (final volume 200 μl) containing 100 mM Bicine–NaOH pH 8.2, 20 mM MgCl_2_, 10 mM NaHCO_3_, 20 mM KCl, 5 mM DTT, 2 mM ADP, 0.2 mM 2,3-dPGA, 0.4 mM NADH, 5 U ml^−1^ enolase, 3.75 U ml^−1^ dPGM, approximately 12.5 U ml^−1^ PK–LDH, and 0.6 mM RuBP.

The assay buffers for the NADH-linked assays were prepared in advance, snap-frozen in aliquots, and kept at −80 °C. Each assay buffer aliquot was only thawed once, as repeated freeze-thawing can result in degradation of the coupling enzymes. Stock solutions and the assay buffer were thawed on ice. The assay buffer was kept in dark on ice before the assay as NADH is light sensitive.

The solubility of the different chemicals was checked beforehand by consulting the manufacturer’s website (for more details see protocols.io dx.doi.org/10.17504/protocols.io.bf8djrs6). Supplementary Fig. S2 shows the significant change in the absorbance of a 0.4 mM NADH solution (final concentration in the different assays mix) at 340 nm when prepared from different NADH stock solutions (0.5 M or 14 mM), highlighting the importance of a correct NADH quantification in the stock solution by checking the concentration of the reduced form through the absorbance at 340 nm and the respective extinction coefficient.

For the photometric assays, the reaction proceeded for at least 2 min to obtain a clear slope of decreasing absorbance. The consumption of RuBP was calculated from the change in absorbance according to:

$$\begin{array}{l} RuBP\;consumption=\frac{Slope\times 0.2}{6.22\times NADH\;factor\times Pathlength}, \end{array}$$

where the slope represents the absorbance change per minute at 340 nm (due to NADH oxidation) in the first minute of the linear range of the activity; 0.2 is the final assay volume (ml); 6.22 is the extinction coefficient of NADH in μmol^−1^ ml cm^−1^; NADH factor is used to account for the number of molecules of NADH oxidized per molecule of RuBP consumed (four for GAPDH–GlyPDH, two for PEPC–MDH and PK–LDH); and pathlength is the correction for the microtiter plate well containing the assay buffer in cm (see Supplementary Fig. S3). 

Measured absorbance values in a microtiter plate need to be normalized to a 1 cm pathlength, which would be found in a typical cuvette used in spectrophotometers (Burnett, 1972). Measurements are corrected using Lambert–Beer’s Law and considering both the volume in each well and the specific well dimensions for each type of microtiter plate. Modern microtiter plate readers frequently include a pathlength correction option, but this feature normally does not consider the properties of the solution. It is important to use the respective assay buffer in determining the pathlength correction factor as the meniscus will affect the pathlength and absorbance reading in the microtiter plate (Lampinen *et al.*, 2012). For the assays used in the present article, there was no statistical difference between the three assay mixtures (see Supplementary Fig. S3), and therefore the average pathlength (5.47 mm) was used for RuBP consumption calculation (Sales *et al.*, 2018).

Rubisco activity was subsequently calculated as described in Sales *et al.* (2018).

### Response to 3-PGA and RuBP concentrations in the photometric assays

Reactions were performed at 25 °C. For the 3-PGA response curves, the assay buffers were as above without RuBP. Initial absorbance at 340 nm was measured before adding 3-PGA. The reaction was initiated by adding 3-PGA to six concentrations (0–100 μM for GAPDH–GlyPDH; 0–200 μM for PEPC–MDH and PK–LDH); the absorbance was monitored until the reaction reached completion and the final absorbance recorded. The total change in absorbance was determined (∆Abs_340nm_) and a linear regression between 3-PGA concentration and ∆Abs_340nm_ calculated.

For the RuBP response curves, the assay buffers were prepared as above. After adding assay buffers to the wells, fully activated Rubisco (ECM) was added to 15 μg ml^−1^ and the reaction started by adding RuBP to six concentrations (0–100 μM for GAPDH–GlyPDH; 0–200 μM for PEPC–MDH and PK–LDH). Rubisco activity was calculated as above. Rubisco quantity was measured by the ^14^C-CABP binding assay (Whitney *et al.*, 1999).

### Data analysis

Statistical analyses were performed in RStudio (version 1.1.453; RStudio Team, 2015). Bar charts and scatterplots were prepared using ‘ggplot2’ (Wickham, 2006). Linear models were fitted to the 3-PGA response curves and to assess the relationship between Rubisco activity measured with photometric and radiometric assays using RStudio. Rubisco Michaelis–Menten constants for RuBP (*K*_m_^RuBP^) and maximum carboxylation rates (*V*_cmax_) were estimated using the Michaelis–Menten equation, which was modelled using the two-parameter model (MM.2) in the ‘drc’ R package (Ritz *et al.*, 2015), which gives identical results to the Lineweaver–Burk method. Statistical significance of differences between means of Rubisco activity measured using each assay was tested using one- and two-way analysis of variance (ANOVA). Where significant main effects of assay and treatment were observed, a Tukey *post hoc* test was used for multiple pairwise comparisons. Pearson’s correlation coefficients (*r*) was computed and visualized in RStudio using the packages ‘Hmisc’ (Harrell, 2019) and ‘corrplot’ (Wei and Simko, 2017).

## Results

### NADH-linked assays underestimate 3-PGA production

**Fig. 2.**
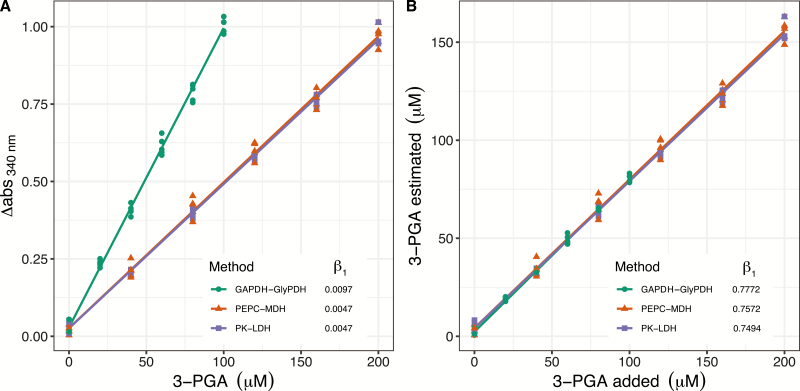
3-Phosphoglycerate (3-PGA) response curves. Change in NADH absorbance at 340 nm (difference between final and initial value, ∆abs_340nm_) in response to increasing 3-PGA concentration (A); and relationship between 3-PGA added and estimated according to the ∆abs_340nm_ obtained in (A) (B), using the GAPDH–GlyPDH, PEPC–MDH, and PK–LDH microtiter plate-based assays. β_1_ is the slope obtained from a fitted linear regression considering the average of the technical replicates (*n*=5).

The NADH-linked assays for measuring Rubisco activity are based on enzymatic reactions that couple 3-PGA formation to NADH oxidation. To determine the accuracy and reliability of each assay in determining the amount of 3-PGA produced by Rubisco, standard curves were generated using increasing concentrations of commercial 3-PGA (Fig. 2). While for the PEPC–MDH and PK–LDH methods two NADH are oxidized per RuBP, for GAPDH–GlyPDH four NADH are oxidized (Fig. 2A). As expected, under conditions of a first order reaction (when 3-PGA is limiting NADH oxidation), the GAPDH–GlyPDH assay had a change in absorbance at 340 nm that was on average approximately twice as fast as observed for the other two assays, PEPC–MDH and PK–LDH (slope of 0.0097 for GAPDH–GlyPDH and 0.0047 for PEPC–MDH and PK–LDH), showing that NADH concentration was not limiting even when four molecules are oxidized per CO_2_ fixed by Rubisco.

3-PGA calculated from the change in NADH absorbance at 340 nm was underestimated, with a decreased accuracy at the higher 3-PGA concentrations. The estimated 3-PGA was 94.6±5.0% of the actual value with 20 μM 3-PGA added and 80.6±2.1% with 100 μM 3-PGA added for the GAPDH–GlyPDH method, while it was 85.7±2.0% or 88.0±9.4% with 40 μM 3-PGA added, and 78.1±2.6% or 78.9±2.1% with 200 μM 3-PGA added for the PEPC–MDH and PK–LDH methods, respectively (Fig. 2B).

### Determination of the Rubisco Michaelis–Menten constant for RuBP

**Fig. 3.**
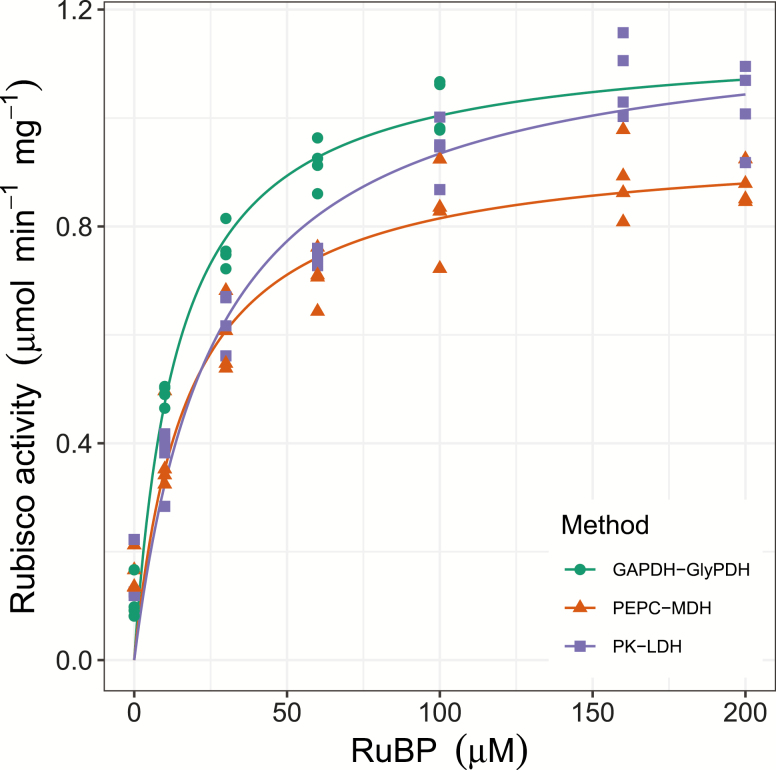
Substrate saturation curve for Rubisco activity measured using the three NADH-linked microtiter plate-based assays: GAPDH–GlyPDH, PEPC–MDH, and PK–LDH. Activity was assayed using Rubisco purified from *Triticum aestivum* cv. Cadenza in the carbamylated form (ECM). Solid lines show Michaelis–Menten two-parameter models based on means of four technical replicates, and symbols show individual data points (*n*=4). The three assays were performed in parallel.

Substrate saturation curves for Rubisco using several concentrations of RuBP revealed differences between the three NADH-linked assays (Fig. 3). The maximum carboxylation activity of Rubisco (*V*_cmax_) estimated from these curves was lower when using the PEPC–MDH assay compared with the GAPDH–GlyPDH and PK–LDH assays (Table 1). The Michaelis–Menten constant for RuBP (*K*_m_^RuBP^) was higher when determined by the PK–LDH assay compared with the GAPDH–GlyPDH assay, but was not significantly different between either of these assays and the PEPC–MDH assay (Tukey HSD).

**Table 1.** Rubisco maximum carboxylation rate (*V*_cmax_) and Michaelis-Menten constant for the substrate RuBP (*K*_m_^RuBP^) determined by the three NADH-linked microtiter plate-based assays: GAPDH–GlyPDH, PEPC–MDH and PK–LDH

Method	*V*_cmax_ (μmol min^–1^ mg^–1^)	*K*_m_^RuBP^ (μM)
GAPDH–GlyPDH	1.15±0.08^a^	14.31±1.27^b^
PEPC–MDH	0.96±0.01^b^	18.08±2.73^ab^
PK–LDH	1.19±0.05^a^	27.29±4.68^a^

Values are means ±SEM (*n*=4 technical replicates) and were estimated by fitting a Michaelis–Menten two-parameter model to the data in Fig. 3. One-way ANOVA showed a significant effect of assay on *V*_cmax_ and *K*_m_^RuBP^ (*P*<0.05). Different letters denote significant differences (Tukey HSD, *P*<0.05).

### The activity of fully carbamylated and inhibited Rubisco purified from wheat

**Fig. 4.**
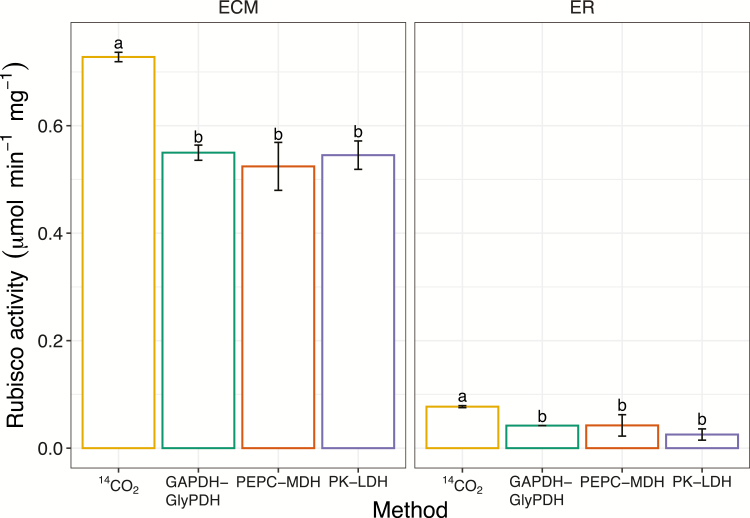
Comparison of assays for measuring activity of purified Rubisco. Fully activated (ECM) and inhibited (ER) Rubisco activities measured by the ^14^CO_2_ fixation assay and by the three NADH-linked microtiter plate-based assays, GAPDH–GlyPDH, PEPC–MDH, and PK–LDH. One-way ANOVA showed a significant effect of the method used on Rubisco activity (*P*<0.05). Different letters denote significant differences (Tukey HSD, *P*<0.05). Values are means ±SEM (*n*=4–5 technical replicates). The percentage of ER in relation to ECM was 5–11%.

To test the reliability of each assay in determining contrasting rates of Rubisco activity, the three NADH-linked assays were used in parallel with the ^14^C-based assay to measure the activity of both fully carbamylated active Rubisco (ECM) and uncarbamylated RuBP-bound inhibited Rubisco (ER) using Rubisco purified from wheat leaves (Fig. 4). Rubisco ECM activities determined with the NADH-coupled assays were approximately 25% lower than the values obtained using the ^14^CO_2_ fixation assay (*P*<0.05). Rubisco ER activities measured using the three NADH-linked assays were also significantly lower than the values obtained using the ^14^CO_2_ fixation assay (*P*<0.05). However, the values determined for both ECM and ER were comparable across the three NADH-linked assays. Rubisco ER activity represented approximately 5–11% of ECM activity using both the three NADH-linked assays and the ^14^C-based assay, which is consistent with previous reports (Carmo-Silva and Salvucci, 2011; Perdomo *et al.*, 2019).

### Rubisco initial and total activities in leaf extracts

**Fig. 5.**
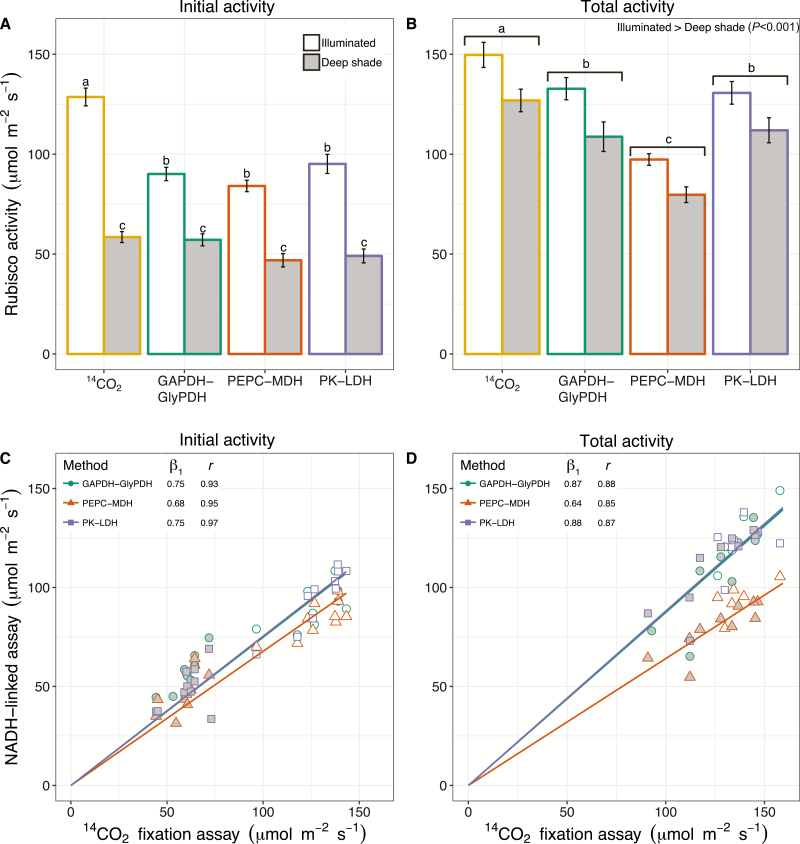
Comparison of assays for measuring Rubisco activity in leaf extracts. (A, B) Rubisco initial (A) and total (B) activity in fully illuminated (white) and deep shaded leaves (grey) measured by the ^14^CO_2_ fixation assay and the three NADH-linked microtiter plate-based assays: GAPDH–GlyPDH, PEPC–MDH, and PK–LDH. Values are means ±SEM (*n*=4–5 technical replicates). One-way ANOVA showed a significant effect of assay and leaf illumination/shading on Rubisco activity (*P*<0.05), with a significant interaction of effects for Rubisco initial activity only. Different letters denote significant differences between each measurement (A) or between assay type (B) (Tukey HSD, *P*<0.05). (C, D) The relationship between Rubisco initial (C) and total (D) activities determined using the three NADH-linked assays and the ^14^CO_2_ fixation assay in fully illuminated (white symbols) and deep shaded leaves (grey symbols). Symbols represent individual measurements and lines represent fitted linear regressions (*n*=19–20 biological replicates). β_1_ is the slope obtained from the linear regression and *r* is the Pearson product-moment correlation coefficient showing the strength of pairwise linear correlations between the ^14^CO_2_ fixation assay and the three NADH-linked assays for measuring Rubisco activity (*P*<0.05).

Consistent with the results obtained using purified Rubisco, the initial activity of Rubisco in wheat leaf extracts prepared from illuminated leaves (PPFD=625±121 μmol photons m^−2^ s^−1^) was ~30% lower with the three NADH-linked assays compared with the ^14^CO_2_ fixation assay (Fig. 5A). The initial activity reflects the physiological activity of Rubisco, representing the active sites that are carbamylated and free from inhibitors in the leaf. Consequently, Rubisco initial activity was considerably lower in leaf extracts prepared from leaves exposed to deep shade (PPFD=5±6 μmol photons m^−2^ s^−1^), regardless of the assay used. The difference between the spectrophotometric and radiometric assays was less clear and not statistically significant when using leaf extracts prepared from leaves exposed to deep shade.

The total activity of Rubisco determined after enabling carbamylation of available active sites by incubation with CO_2_ and Mg^2+^ showed less clear differences between assays and between illuminated versus deep shade leaves (Fig. 5B). There was a significant effect of light treatment and assay (*P*<0.001), but no significant interaction of effects (*P*=0.934). The lower total activity of Rubisco in deep shade leaves would suggest that inhibitors are blocking active sites (Parry *et al.*, 2008), yet the difference between shaded and illuminated leaves was not always significant. Rubisco total activities determined with the PEPC–MDH assay were lower than the values obtained with all other assays, suggesting that when using PEPC and MDH as coupling enzymes, MgCl_2_ and NaHCO_3_ concentration might be limiting the speed of the reaction especially when Rubisco is most active (total activity versus initial activity).

Considering all the samples from illuminated and deep shade conditions, the three NADH-linked assays correlated strongly and similarly with the ^14^C-based assay (*P*<0.001; Fig. 5C, D). All three assays showed a less pronounced increase in Rubisco activity between shaded and illuminated leaves than observed for the ^14^C-based assay, as shown by slopes (β _1_) lower than 1. Even though the fitted linear regressions were similar for the three NADH-linked assays, the PK–LDH and GlyPDH–GAPDH assays were more closely associated with the radiometric assay (β _1_=0.75–0.88) than the PEPC–MDH assay (β _1_=0.64–0.68).

The activation state of Rubisco, i.e. comparing the initial activity measured immediately in leaf extracts with the activity of fully carbamylated enzyme (Perchorowicz *et al.*, 1981; Scales *et al.*, 2014), was not estimated correctly by the NADH-based assays when compared with the radiometric methods (data not shown). The total activities (Fig. 5B, D) showed a different pattern compared with the initial activity (Fig. 5A, C), compromising the activation state estimation.

## Discussion

Rubisco activities measured by three NADH-linked microtiter plate-based assays correlated strongly with values obtained by the ^14^C-based assay, but the absolute values were ~30% lower. The assay using PK–LDH as the coupling enzymes showed a strong correlation with the radiometric assay and was most economical, making it the most suitable assay for large phenotyping projects to identify genetic variation in Rubisco activity, providing reliable results and higher throughput than the ^14^C-based assay. We discuss a few considerations to maximize the reliability of this assay and argue that NADH-linked assays are not as reliable as the ^14^C-based assay for measuring Rubisco initial activity when the carbamylation of the enzyme in leaves is low, e.g. in shaded leaves.

### NADH-linked microtiter plate-based assays underestimate 3-PGA formation

3-PGA concentration was calculated from the change in NADH absorbance at 340 nm (Fig. 2). The three microtiter plate-based NADH-linked assays underestimated 3-PGA formation, with accuracy decreasing at the higher 3-PGA concentrations. When 20 μM 3-PGA was added, the estimated 3-PGA was 94.6±4.4% of the actual value, while it was 75.9±2.5% with 400 μM 3-PGA added (Fig. 1B). These results highlight the importance of using a suitable amount of Rubisco (or leaf extract), to ensure a sub-saturating concentration of 3-PGA, for accurate estimation of NADH oxidation (Sales *et al.*, 2018). If large variation in leaf sample sizes and/or Rubisco activities is expected, the NADH-linked assays are unreliable as the amount of 3-PGA formed during the reactions will vary and the comparison between samples will be prone to error.

The substrate saturation curves for Rubisco (Fig. 3) showed that the results obtained with the three NADH-linked assays are different. The lower *V*_cmax_ determined with the PEPC–MDH assay compared with the GAPDH–GlyPDH and PK–LDH assays suggests that the PEPC–MDH assay limited the maximum measurable rate for Rubisco activity. *K*_m_^RuBP^ values estimated by the PK–LDH and GAPDH–GlyPDH assays were higher than those found with PEPC–MDH, and closer to previously reported values of *K*_m_^RuBP^ for wheat (31±4 μM; Yeoh *et al.*, 1981).

### The activity of purified Rubisco measured using NADH-linked microtiter plate-based assays is lower than with the ^14^C-based assay

The use of purified Rubisco in the fully carbamylated (ECM) and inhibited (ER) form to test the different assays is important for two main reasons: first, to have isolated Rubisco and make sure that no other components present in the leaf extract interfere with the reaction; and second, to have contrasting slope ranges and verify that the different methods can detect these differences. Rubisco ECM activities measured using the three NADH-linked microtiter plate-based assays were approximately 25% lower than the values obtained using the ^14^CO_2_ fixation assay (Fig. 4), which is consistent with previous studies comparing spectrophotometric and radiometric methods (Sharwood et al., 2008, 2016*b*). In NADH-based methods, it is important to have a good slope of decreasing absorbance (not too steep and not too shallow) for accurate estimation of NADH oxidation. Measurements of ER activities had an absorbance change per minute at least 10 times smaller than ECM samples, and ER activities determined by the NADH-linked methods were still lower than the values obtained using the ^14^C-based assay, indicating that the rate of NADH consumption is not the cause of the lower results found using NADH-linked assays.

NADH-linked assays rely on a long reaction chain, and it is unlikely that the same absolute values would be observed between measurements based on a single reaction step in the ^14^CO_2_ fixation assay and the multi-step NADH-linked assays. Characterization of Rubisco catalytic properties requires careful experimentation, and discrepancies between laboratories and the different methods used for estimating Rubisco carboxylation rates have been discussed before (Galmés *et al.*, 2006; Silva-Pérez *et al.*, 2017).

### NADH-linked microtiter plate-based assays can be used reliably to phenotype Rubisco activity in leaf extracts, except when carbamylation is low

Phenotyping Rubisco activity for crop improvement efforts is important as it is well known that improving Rubisco can contribute to increase photosynthetic efficiency in crop plants (Long *et al.*, 2015; Betti *et al.*, 2016). A cheaper and higher throughput method than the radiometric assay is desirable, but it is important to have accurate methods for this purpose. Rubisco activities in fully illuminated and shaded leaves were used here to test whether the three NADH-linked microtiter plate-based assays provide comparable results to those obtained with the radiometric assay. The three NADH-linked assays were successful at discerning contrasting rates of Rubisco activity, i.e. in ECM and ER Rubisco (Fig. 4), but to what extent can the different methods distinguish Rubisco activities in fully illuminated and shaded leaves?

All three NADH-linked microtiter plate-based assays effectively distinguished Rubisco initial (Fig. 5A) and total activities (Fig. 5B) in illuminated leaves, but NADH-linked assays failed to measure Rubisco initial activity accurately when the carbamylation of the enzyme in the leaves was very low (e.g. deep shaded leaves). The lack of statistically significant differences in Rubisco initial activity of shaded leaves between the ^14^C-based assay and the NADH-linked assays (Fig. 5A) is probably due to the longer duration of the NADH-linked assays (typically 2 min from starting to pipet the leaf extract to obtaining the slope) compared with the ^14^C-based assay (typically 30–60 s). During the NADH-linked assays, some Rubisco active sites are likely to become carbamylated as the leaf extract is exposed to high CO_2_ and Mg^2+^ in the assay buffer. Therefore, these assays would not be suitable for measuring Rubisco initial activity and/or activation state at different levels in the canopy or in conditions in which low intercellular CO_2_ (*c*_i_) is promoted (e.g. low light, drought stress, cold stress); the 30 s ^14^C-based assay is recommended in such situations. The same result was not observed for the ER activity estimated using purified Rubisco by NADH-linked assays, which was lower than the ^14^C-based assay (Fig. 4), because the active sites of Rubisco were blocked tightly. Conversely, in shaded leaves, many of the Rubisco active sites could be uncarbamylated and free from RuBP, and therefore available for rapid carbamylation during the Rubisco initial activity assay.

In deep-shaded leaves exposed to very low light levels, it is likely that some Rubisco active sites would have been blocked by the tight-binding of inhibitory compounds. In this situation, Rubisco will not be effectively carbamylated during incubation with saturating concentrations of CO_2_ and Mg^2+^ in the assay (Salvucci and Anderson, 1987; Sage *et al.*, 1988; Parry *et al.*, 1997), resulting in consistently lower total activity values in shaded compared with illuminated leaves (Fig. 5B).

Importantly, when considering large phenotyping efforts to identify genetic variation in Rubisco activity in illuminated leaves, the NADH-linked assays provide a higher throughput alternative to the ^14^C-based assay that is more likely to be adaptable to any laboratory situation. Particularly when Rubisco total activity is the target, the GAPDH–GlyPDH and PK–LDH assays would be suitable and highly recommended.

### Important considerations for using NADH-linked microtiter plate-based assays to measure Rubisco activity

In the ^14^C-based radiometric assay, one step reaction enables the measurement of incorporation of ^14^CO_2_ into [^14^C]3-PGA. Conversely, the NADH-linked spectrophotometric assays depend on a range of chemicals and reactions to link 3-PGA formation to NADH oxidation. Some care is required with these NADH-linked assays to minimize inconsistencies and maximize their reliability. One important consideration is the pathlength correction as measured absorbance in microtiter plates needs to be normalized to a 1 cm pathlength, and this normalization will depend on the microtiter plate type and solution used. Other considerations include the solubility of the different chemicals used to prepare the assay buffer, the light sensitivity of NADH, and clearly defined slopes. It is essential to ensure thorough homogenization of each solution added to the assay buffer in the wells of the microtiter plate to reduce variability between replicates, while also ensuring that no bubbles are added in the solution.

Table 2 shows some factors to be considered when choosing between the ^14^C-based assay and the different NADH-linked assays. The choice will depend on the available resources, e.g. facilities for working with radioactivity, homemade chemicals such as dPGM, and research project objectives. For example, if precise temperature control is required, probably the microtiter plate assay methods will not be adequate as microtiter plate readers frequently lack the ability to precisely control the temperature, especially below room temperature. The radiometric assay can be performed in a water bath or temperature block, which enables a precise control of temperatures of the assays. It is essential to keep in mind that the ^14^C-based assay is the most precise method for measuring Rubisco activity when absolute values are the goal. However, others have shown that in assays performed at higher temperatures, the CO_2_ concentration may become sub-saturating, affecting the precision of the ^14^C-based assay (Boyd *et al.*, 2019). NADH-linked assays are cheaper (see Supplementary Table S1) and produce less lab waste, but have more variability and less precision than the ^14^C-based assay.

**Table 2.** Considerations for choosing the Rubisco activity assay method most suitable to each research situation

Factor to be considered	Method			
	^14^CO_2_-based assay	Microtiter-based assay		
		GAPDH–GlyPDH	PEPC–MDH	PK–LDH
Suitable for determination of absolute Rubisco activity values	✓	×	×	×
Suitable for measuring Rubisco activation state	✓	×	×	×
Suitable for measuring Rca activity	✓	×	✓	×
Large variation expected in leaf samples to be tested	✓	×	×	×
No requirement for producing dPGM	✓	✓	×	×
Precise temperature control	✓	×	×	×
Immediate visualization of results	×	✓	✓	✓
Higher throughput	×	✓	✓	✓
Lower cost per sample	×	×	×	✓
Lower lab waste and environmental impact	×	✓	✓	✓

Rubisco activase (Rca) activity can be measured by its ability to increase the activity of Rubisco. Rca exhibits a low affinity for ATP and a very high affinity for inhibition by ADP. For this reason, Rca activity can only be measured by NADH-linked assays that do not require ADP in the assay mix. Within the three NADH-linked assays tested here, PEPC–MDH does not require ADP, and therefore it is suitable for measuring Rca activity. For more details, see Scales *et al.* (2014).

## Conclusion

The three NADH-linked microtiter plate-based assays retrieve lower values of Rubisco activity compared with the ^14^CO_2_ fixation assay, yet all three assays correlated strongly with the radiometric assay, with the PK–LDH assay showing the overall strongest correlation. The maximum TSP in leaf extracts used for NADH-linked assays should be ~3.5 mg ml^−1^ for C_3_ plants and double for C_4_ species. PEPC–MDH would be a suitable option for situations in which ADP/ATP might interfere with the assay, but more tests are required to understand the lower values found with this method. The GAPDH–GlyPDH method proved more laborious than the other methods. Provided that a few considerations are taken into account, we would recommend the microtiter plate-based PK–LDH assay as a reliable, cheaper, and higher throughput method to phenotype Rubisco activity in illuminated leaves for crop improvement efforts. For a more thorough characterization of Rubisco properties requiring the measurement of absolutes rates of carboxylation and assessment of carbamylation levels, the single-reaction ^14^C-based method must be used.

## Supplementary data

Supplementary data are available at *JXB online*.

Fig. S1. Linear increase in RuBP consumption with total soluble protein concentration (TSP) in the leaf extract.

Fig. S2. Absorbance at 340 nm of NADH-linked assay mixtures prepared using two different NADH stock solutions.

Fig. S3. Pathlength correction for absorbance values of the three NADH-linked assay mixtures measured in a flat-bottom 96-well microtiter plate.

Table S1. Estimated costs of the radiometric (^14^CO_2_) and NADH-linked microtiter plate-based assays (GAPDH–GlyPDH, PEPC–MDH, PK–LDH) for measuring Rubisco

activity.

eraa289_suppl_Supplementary_Materials

## Data Availability

The data presented in this publication are available at the data repository used by Lancaster University: https://dx.doi.org/10.17635/lancaster/researchdata/366.

## Abbreviations

1,3-PGA: 1,3-diphosphoglycerate
2-PGA: 2-phosphoglycerate
2,3-dPGA: 2,3-diphospho-D-glyceric acid
3-PGA: 3-phosphoglycerate
DHAP: di-hydroxyacetone phosphate
dPGM: 2,3-dPGA-dependent phosphoglycerate mutase
ECM: Rubisco carbamylated with bound Mg^2+^
ER: uncarbamylated Rubisco complexed with RuBP
G3P: glyceraldehyde-3-phosphate
GAPDH: glyceraldehyde-tr3-phosphate dehydrogenase
Gly3P: glycerol 3-phosphate
GlyPDH: glycerolphosphate dehydrogenase
LDH: lactate dehydrogenase
MDH: malate dehydrogenase
*K*_m_^RuBP^: Michaelis–Menten constant for RuBP
OAA: oxaloacetate
PEP: phosphoenolpyruvate
PEPC: phosphoenolpyruvate carboxylase
PGK: 3-phosphoglycerate kinase
PK: pyruvate kinase
PPFD: photosynthetic photon flux density
RuBP: ribulose-1,5-bisphosphate
TPI: triosephosphate isomerase
TSP: total soluble protein
*V*_cmax_: maximum Rubisco carboxylase activity estimated by Michaelis–Menten fitting
