# Supplementary material for: Measuring Rubisco activity: challenges and opportunities of NADH-linked microtiter plate-based and 14C-based assays
Source: J Exp Bot. 2020 Jun 30;71(18):5302–12. doi: 10.1093/jxb/eraa289 (PMC7501812; doi:10.1093/jxb/eraa289)
Supplement: eraa289_suppl_Supplementary_Materials [file eraa289_suppl_supplementary_materials.pdf]

Measuring Rubisco activity: challenges and opportunities of NADH-linked microtiter plate-based and ^{14}C-based assays

Cristina R. G. Sales[1,*], Anabela Bernardes da Silva[2], Elizabete Carmo-Silva[1,*]

* To whom correspondence may be addressed. Email: c.r.sales@lancaster.ac.uk or e.carmosilva@lancaster.ac.uk

Supplementary information

Table S1. Costs of radiometric and NADH-linked assays for Rubisco activity.
Figure S1. Linear increase in RuBP consumption with total soluble protein concentration (TSP) in the leaf extract.
Figure S2. Absorbance of NADH prepared using different stock solutions.
Figure S3. Pathlength correction for absorbance measured in a microtiter plate.

Table S1. Estimated costs of the radiometric ($^{14}CO_2$) and NADH-linked microtiter plate-based assays (GAPDH-GlyPDH, PEPC-MDH, PK-LDH) for measuring Rubisco activity.

	**Cost per leaf sample (£)**			
	$^{14}CO_2$	GAPDH-GlyPDH	PEPC-MDH	PK-LDH
Extraction buffer	0.77	0.77	0.77	0.77
Assay mix	10.05	5.05	3.31	2.71
Total	11.82	5.82	4.08	3.48

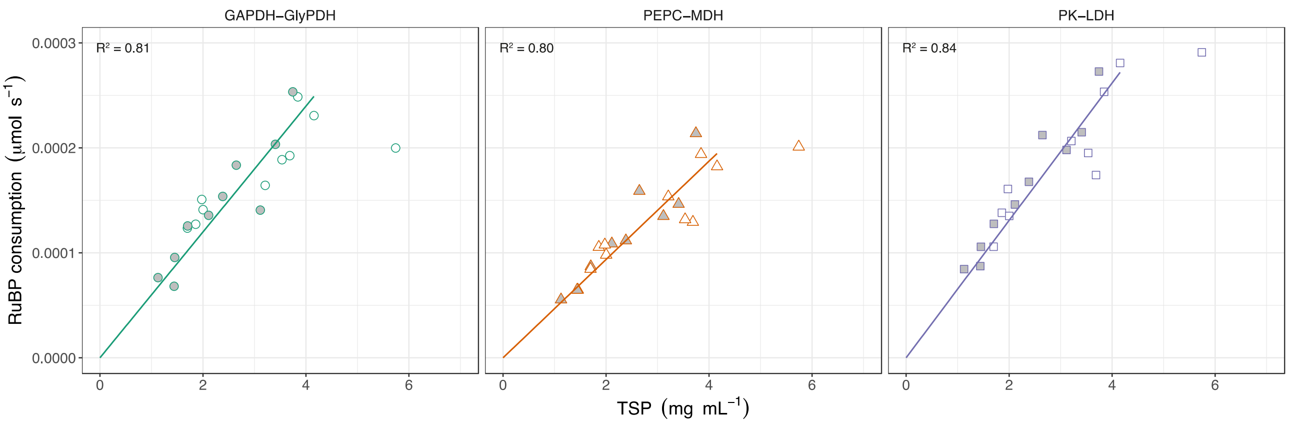

Fig. S1. Linear increase in RuBP consumption with total soluble protein concentration (TSP) in the leaf extract. Data points correspond to individual assays of Rubisco total activity in fully illuminated (white) and shaded leaves (grey) measured by the three NADH-linked microtiter plate-based assays: GAPDH-GlyPDH, PEPC-MDH and PK-LDH. The three assays were performed in parallel for each sample. Symbols represent individual measurements and lines represent fitted linear regressions (*n* =19–20 biological replicates). The data point corresponding to 6 mg mL^{-1} TSP provides a good example of saturation of the assay: the activity of Rubisco is too fast and the NADH concentration available limits the reaction of the coupling enzymes (this data point was considered an outlier and not included in the dataset used for the linear regressions shown here or the results shown in Fig. 5).

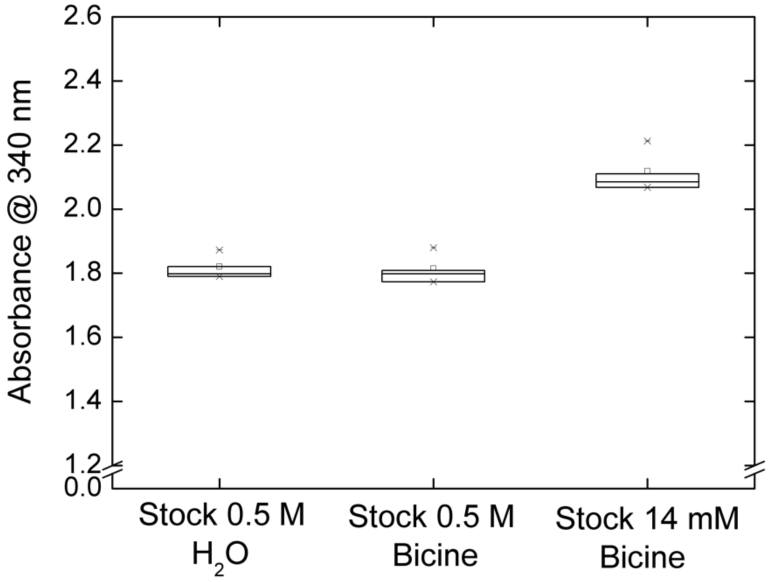

Fig. S2. Absorbance at 340 nm of NADH-linked assay mixtures prepared using two different NADH stock solutions. Results show the importance of preparing a less concentrated NADH stock solution (14 mM) for accurate NADH quantification in microtiter plates by measurement of the absorbance at 340 nm and using the respective extinction coefficient, without requiring large dilutions (e.g., >1500 fold with 0.5 M), which are prone to error. The use of NADH stock solutions prepared in H_2O or Bicine did not interfere with the measured absorbance.

A)

$$Pathlength = \frac{A_{975\ (well)} - A_{900\ (well)}}{A_{975\ (cuvette)} - A_{900\ (cuvette)}} \times 10\ mm = \frac{A_{975\ (well)} - A_{900\ (well)}}{K_{factor}} \times 10\ mm$$

$$A_{corrected} = A_{raw} \times \frac{10\ mm}{Pathlength_{(well)}} = A_{raw} \times \frac{K_{factor}}{A_{975\ (well)} - A_{900\ (well)}}$$

B)

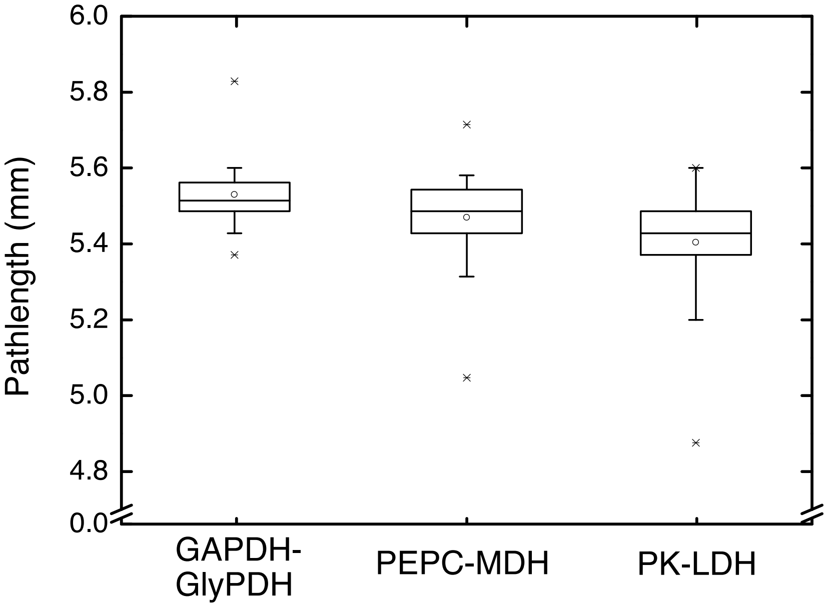

Fig. S3. Pathlength correction for absorbance values of the three NADH-linked assay mixtures measured in a flat-bottom 96-well microtiter plate. A) Equations used for determining the correct pathlength based on Lampinem *et al*. (2012). B) Pathlength value for each of three NADH-linked assay mixture: GAPDH-GlyPDH, PEPC-MDH and PK-LDH (*n*=4 technical replicates). One-way ANOVA showed no significant difference between the three assays (P > 0.05), therefore the average pathlength was used (5.47 mm).
